# An ontology-based search engine for protein-protein interactions

**DOI:** 10.1186/1471-2105-11-S1-S23

**Published:** 2010-01-18

**Authors:** Byungkyu Park, Kyungsook Han

**Affiliations:** 1 School of Computer Science and Engineering, Inha University, Incheon 402-751, South Korea

## Abstract

**Background:**

Keyword matching or ID matching is the most common searching method in a large database of protein-protein interactions. They are purely syntactic methods, and retrieve the records in the database that contain a keyword or ID specified in a query. Such syntactic search methods often retrieve too few search results or no results despite many potential matches present in the database.

**Results:**

We have developed a new method for representing protein-protein interactions and the Gene Ontology (GO) using modified Gödel numbers. This representation is hidden from users but enables a search engine using the representation to efficiently search protein-protein interactions in a biologically meaningful way. Given a query protein with optional search conditions expressed in one or more GO terms, the search engine finds all the interaction partners of the query protein by unique prime factorization of the modified Gödel numbers representing the query protein and the search conditions.

**Conclusion:**

Representing the biological relations of proteins and their GO annotations by modified Gödel numbers makes a search engine efficiently find all protein-protein interactions by prime factorization of the numbers. Keyword matching or ID matching search methods often miss the interactions involving a protein that has no explicit annotations matching the search condition, but our search engine retrieves such interactions as well if they satisfy the search condition with a more specific term in the ontology.

## Background

Recent advances in high-throughput interaction detection methods such as yeast two-hybrid and mass spectrometry techniques have led to a rapid expansion of protein-protein interaction data in several organisms. For example, there are about 8, 000 interactions between 4, 000 *S. cerevisiae *proteins, approximately 24, 000 interactions between 7, 600 *D. melanogaster *proteins, and over 5, 100 interactions between 2, 900 *C. elegans *proteins [1]. More than 137, 000 protein interactions and 60, 000 genetic interactions are also known for six major model organisms [2]. Several databases have been built for the large amount of protein-protein interaction data, which include BOND [3], DIP [4], MIPS [5], HPRD [6], HPID [7] and BioGRID [2]. Most of the databases allow the user to retrieve protein-protein interactions that satisfy a condition specified in a query. Keyword matching or ID matching is one of the most commonly used searching methods. This type of search retrieves all of the records in the database which contain a keyword or ID specified in a query. The user can alter retrieval results using Boolean operators such as AND, OR and NOT.

However, a search method based on keyword matching or ID-matching is purely syntactic and does not consider biological relations between the keywords or IDs. If the user gets too many protein-protein interactions, selecting the protein-protein interactions to focus on entirely relies on the discretion of the user. If the user gets too few protein-protein interactions or no results at all, the user will probably have to look for other resources. For example, BOND returns 5, 100 protein-protein interactions for a keyword query of 'ATP binding' whereas it returns only 96 interactions for a keyword query of 'nucleotide binding'. The term 'nucleotide binding' is at a higher level than 'ATP binding' in the Gene Ontology (GO) hierarchy [8], but it returns much fewer search results than 'ATP binding'. This search anomaly occurs because the search method of BOND does not consider the biological relation between keywords. Besides, the user must enter an exact keyword or ID in the query since BOND and many other protein-protein interaction databases do not support the 'autocomplete' feature when searching the databases.

Recently a few ontology-based information retrieval methods have developed for biological literature or databases [9-11], but little work has been reported on the ontology-based search for protein-protein interactions in databases. We developed a new representation of the Gene Ontology (GO) and a search engine that finds all the semantically relevant interactions of a query protein using the representation. For a GO term, all the GO terms at the lower level than the GO term in the GO hierarchy are automatically considered when searching for protein-protein interactions. For example, when dealing with a query like "for protein p annotated with a GO term f, find the interaction partners of p", the search engine considers not only the GO annotation f but also all the GO annotations below f in the GO hierarchy. Several computational methods have been developed to elucidate protein function from the analysis of protein-protein interaction data [12], and our search engine will be useful to identify proteins with common function or subcellular localization. This paper presents the development of a new representation method of protein-protein interactions and a search engine for protein-protein interactions using the representation method.

## Methods

Gödel numbers are typically used to uniquely encode any list of positive integers {*a*_1_, *a*_2_, ..., *a*_*n*_} by(1)

where *p*_*k *_is the *k*th prime number [13].

However, the original Gödel numbers defined by equation (1) cannot represent the Directed Acyclic Graph (DAG) structure of the Gene Ontology. Consider an example shown in Table 1, in which a unique natural number represents a term and a Gödel number represents the relation between the terms. Term4 is a kind of Term2 by Relation R3, and Term4 is a kind of Term1 by Relation R1. But these relations cannot be inferred unambiguously from the representation of Table 1.

**Table 1:** Gödel number representation. Original Gödel numbers cannot represent the Directed Acyclic Graph (DAG) structure of the Gene Ontology.

Term	Natural number	Relation	Gödel Number
is a	1		
part of	2	R1: Term2 is a Term1	2^4^3^1^5^3 ^= 6, 000
Term1	3	R2: Term3 is part of Term2	2^5^3^2^5^4 ^= 180, 000
Term2	4	R3: Term4 is a Term2	2^6^3^1^5^4 ^= 120, 000
Term3	5		
Term4	6		

The following example shows why the original Gödel numbers fail to represent the GO structure. Suppose that we represent terms by unique natural numbers and the relations between them by Gödel numbers. In this example, Term4 is a kind of Term2 by relation R3, and Term4 is a kind of Term1 by relation R1. But these relations cannot be inferred from the representation because the original Gödel numbers are not sufficient to represent the DAG structure of GO.

Therefore, we modify the Gödel numbers as follows:

1. Assign each term a prime number instead of a natural number using Algorithms 1 and 2.

2. Represent each relation between the terms by a modified Gödel number using Algorithm 3. The modified Gödel number is the product of the prime numbers representing the terms in the relation and their ancestors, including the root term in the Gene Ontology hierarchy.

For example, relation R3 of Table 2 is represented by 42, which has prime factors of 2 (Term1, root node in the hierarchy), 3 (Term2), and 7 (Term3). This representation enables us to infer the meaning of R3 by unique factorization of R3 into prime numbers representing Term1, Term2, and Term3.

**Table 2:** Modified Gödel number representation.

Term	Prime number	Relation	Modified Gödel Number
Term1	2		
Term2	3	R1: Term2 is a Term1	relation(is a) = 3 × 2 = 6
Term3	5	R2: Term3 is part of Term2	relation(part of) = 5 × 3 × 2 = 30
Term4	7	R3: Term4 is a Term2	relation(is a) = 7 × 3 × 2 = 42

Each term is assigned a prime number instead of natural number, and each relation is denoted by a modified Gödel number, which is a multiplication of prime numbers representing the term and its ancestors in the ontology hierarchy. For example, relation R3 is denoted by 42, which has prime factors 2 (Term1, root node in the hierarchy), 3 (Term2), and 7 (Term3). Using this representation, relation R3 can be easily inferred by unique factorization of it into primes (Term1, Term2, and Term3).

Table 3 shows an example of predicting protein-protein interactions from domain-domain interactions using the representation. Suppose that domainA interacts with domainB (R4 in Table 3), proteinA has domainA (R5), and that proteinB has domainB (R6). These relations can be represented by modified Gödel numbers, as shown in Table 3. Using this representation, the hypothesis that proteinA interacts with proteinB can be tested by simple arithmetic operations such as integer division and modulo operation.(2)(3)(4)

**Table 3:** Reasoning protein-protein interactions.

Term	Prime number	Relation	Modified Gödel Number
domainA	11		
domainB	13	R4: domainA interacts with domainB	relation(interacts) = 11 × 13 = 143
ProteinA	17	R5: ProteinA has domainA	relation(has a) = 17 × 11 = 187
ProteinB	19	R6: ProteinB has domainB	relation(has a) = 19 × 13 = 247

Using our representation, it is possible to infer protein-protein interactions from domain-domain interactions. Suppose that domainA interacts with domainB, proteinA has domainA, and that proteinB has domainB. Simple arithmetic operations such as integer division and modulo operations are sufficient to infer that ProteinA interacts with ProteinB.

In this example, the relation that domainA interacts with domainB is represented by a modified Gödel number 143, which is the product of 11 (representing domainA) and 13 (domainB) (equation 2). The hypothesis that proteinA interacts with proteinB is represented by a modified Gödel number 46, 189 from the multiplication of 187 (proteinA) by 247 (proteinB) (equation 3). Since the remainder after dividing 46, 189 (representing the hypothesis that proteinA interacts with proteinB) by 143 (representing the relation that domainA interacts with domainB) is 0 (equation 4), the hypothesis that proteinA interacts with proteinB turns out to be true.

**Algorithm 1 **Generate modified Gödelnumbers

This algorithm assigns prime numbers to GO terms, stores the relation of the GO terms in a local DB by calling Algorithm 2, and generates the modified Gödel numbers by multiplying the prime numbers.

1: *T *= {*t*_1_, *t*_2_, ..., *t*_*G*_} {*G *is the number of GO terms.}

2: *P *= {*p*_1_, *p*_2_, ..., *p*_*G*_} {*P *is an ordered set of prime numbers.}

3: **for all ***i *∈ {1, 2, ..., *G*} **do**

4:   *t*_*i *_← *p*_*i *_{Assign a prime number to a GO term.}

5: **end for**

6: **for all ***t *∈ {*t*_1_, *t*_2_, ..., *t*_*G*_} **do**

7:   Algorithm2(*t*. *key*, *t*) {Store the prime number assignment in a local DB.}

8: **end for**

9: **for all ***t *∈ {*t*_1_, *t*_2_, ..., *t*_*G*_} **do**

10:   Modified Gödel number ← Algorithm3(*t*. *key*)

11: **end for**

**Algorithm 2 **StoreRepresentation(*Term.key*, *Term*)

This algorithm stores the prime number assignment of the GO term and its parents by a recursive call until the parameter Term is a root term of the hierarchy.

1: **if ***Term.isRoot *== false **then**

2:   **for all ***t *∈ *Term.parent ***do**

3:      SetRelation(*Term.key*, *t.key*, *t.prime*) {Store the prime numbers assigned to *Term *and its parents.}

4:      Algorithm2(*Term.key*, *t*) {Recursive call for the parent of *Term*}

5:   **end for**

6: **end if**

**Algorithm 3 **ModifiedGödelNumber(*Term.key*)

This algorithm calculates a modified Gödel number by multiplying the prime numbers representing the parameter Term.key and its ancestor terms in the ontology hierarchy.

1: ArrayList *list *= GetRelation(*Term.key*) {Retrieve the prime numbers for *Term *and its ancestors.}

2: *var *= 1

3: **for all ***t *∈ *list ***do**

4:   *var *= *var *× *t.prime*

5: **end for**

6: **return ***var*

## Results and discussion

### User interface of the search engine

**Figure 1:**
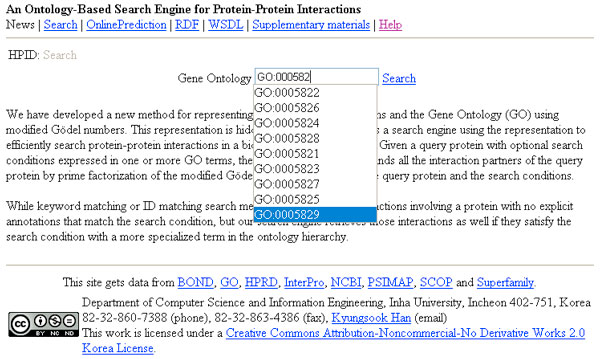
**User interface of the ontology-based search engine**. An example of using the autocomplete functionality for GO terms.

A prototype of the ontology-based search engine has been implemented in the C# programming language [21]. We generated more than 26, 000 prime numbers using the Sieve of Eratosthenes [14], and used the Java BigInteger class to store the numbers and to perform multiplication and modulo operations on them. When the user specifies a GO term or protein superfamily [15] for the query protein, the search engine returns all interactions that involve the protein annotated with the GO term or superfamily as well as the proteins annotated with more specific terms than the specified GO term. To make the search engine easy to use, it provides autocomplete functionality for GO terms or protein superfamilies. So, a partial term entered by the user is expanded into one or more complete GO terms or superfamilies that are consistent with the partial term. An example of using the autocomplete functionality for GO terms in the search engine is shown in Figure 1.

The user can also get protein-protein interactions from the web service of the search engine using the Web Services Description Language (WSDL). WSDL is an XML format for describing network services as a set of endpoints operating on messages containing either document-oriented or procedure-oriented information [16]. The interaction data returned by the search engine can be displayed and saved in the PSI-MI format [17] or in the PSI-MI format with XML style sheets.

### Comparison of the search methods

We tested the ontology-based search engine on the interaction data of HPRD [6] and compared it with the ID-matching search method. Table 4 shows the number of protein-protein interactions found in HPRD by the two search methods. HPRD release 7 contains 38, 190 interactions between 8, 800 human proteins, which are annotated with 470 GO terms. The total number of GO terms is more than 26, 000, but only 1.8% of the 26, 000 GO terms are used for annotating human proteins in HPRD. Our search engine can infer up to 698 GO terms for human proteins from the 470 GO terms that were used for annotating human proteins (see the supplementary material at [21]).

**Table 4:** Comparison of search results by two search methods.

GO term ID	GO term name	ID-matching search	Ontology-based search
Biological process			
GO:0008150	biological process	5 (0.01%)	36, 523 (95.63%)
GO:0008152	metabolic process	2, 862 (7.49%)	19, 828 (51.92%)
GO:0044238	primary metabolic process	0 (0.00%)	17, 434 (45.65%)
GO:0043170	macromolecule metabolic process	0 (0.00%)	7, 211 (18.88%)
GO:0019538	protein metabolic process	5, 324 (13.94%)	5, 659 (14.82%)

Molecular function			
GO:0003676	nucleic acid binding	10 (0.03%)	8, 733 (22.87%)
GO:0003677	DNA binding	1, 944 (5.09%)	6, 935 (18.16%)
GO:0003700	transcription factor activity	5, 164 (13.52%)	5, 164 (13.52%)

Cellular component			
GO:0005622	intracellular	0 (0.00%)	31, 694 (82.99%)
GO:0005737	cytoplasm	17, 312 (45.33%)	20, 990 (54.96%)
GO:0005829	cytosol	452 (1.18%)	452 (1.18%)

The number of protein-protein interactions found in HPRD release 7 by each search method. The numbers inside parentheses indicate the ratio of the interactions to the total 38, 190 interactions of HPRD. The ID-matching search often finds more interactions with a specialized GO terms than with a less specialized terms since it does not consider semantic relation between ontology terms.

**Figure 2:**
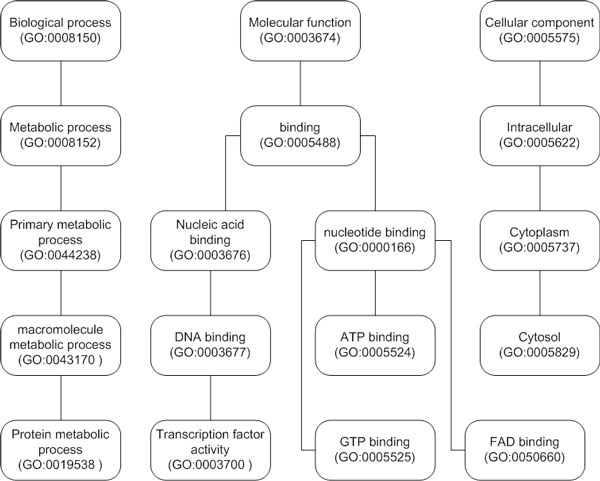
**Example of the gene ontology hierarchy**. A partial view of the three ontologies of the Gene Ontology (GO). The GO terms 'biological process', 'molecular function', and 'cellular component' are the root nodes of three GO hierarchies. Several intermediate terms between the nodes are not shown for clarity.

The GO term ID of GO:0008150 is the root node of the GO hierarchy for biological process. With a query of GO:0008150, the ontology-based search engine found 36, 523 interactions (95.6% of the total 38, 190 interactions of HPRD), but the ID-matching search retrieved only 5 interactions (0.01% of the total 38, 190 interactions of HPRD). With a query of GO:0008152 for metabolic process, which is the descendent node of GO:0008150 in the GO hierarchy (Figure 2), the ontology-based search engine found 19, 828 interactions (51.9% of the total 38, 190 interactions of HPRD), but the ID-matching search found 2, 862 interactions (7.5% of the total 38, 190 interactions). The ID-matching search returned more search results with a more specific term than with a less specific one. The ID-matching search found no interactions with a query of GO:0044238 or GO:0043170, but found 5, 324 interactions with a query of GO:0019538, which is at a lower level than GO:0044238 or GO:0043170. These search anomalies occur because the ID-matching search method does a purely syntactic search and does not consider the relation of GO terms at all. In contrast, the ontology-based search finds interactions not only by the GO term specified in the query but by specialized terms of the term.

**Figure 3:**
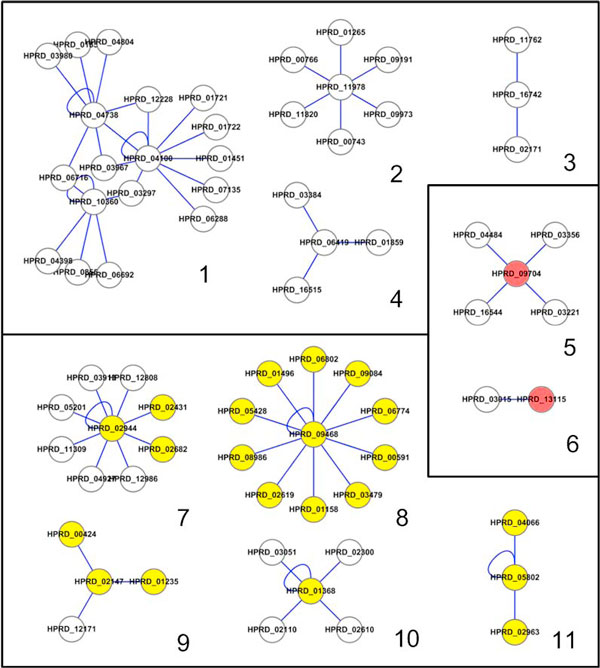
**Interaction network of human proteins found with Nucleotide binding and ATP binding**. Networks 1-11 represent the 70 protein-protein interactions found by the ontology-based search with the query of 'Nucleotide binding' from the HPRD data. Networks 7-11 represent the 31 interactions found by the ontology-based search with the query of 'ATP binding', which is a more specific term than 'Nucleotide binding'. The ID-matching search found only 5 interactions (networks 5-6) with 'Nucleotide binding' and missed all the other interactions whereas its search results with 'ATP binding' are same as those of the ontology-based search (networks 7-11). Yellow nodes represent the human proteins explicitly annotated with 'ATP binding', pink nodes represent the human protein explicitly annotated with 'Nucleotide binding', and white nodes represent the human proteins with no explicit annotation of 'ATP binding' nor 'Nucleotide binding'. The GO term IDs of the proteins found by the search methods are listed in Table 5.

Figure 3 shows the interaction networks of human proteins, which were found by the two search methods and visualized by Cytoscape [18]. Networks 1-11 of Figure 3 represent the 70 protein-protein interactions found by the ontology-based search with the query of 'Nucleotide binding' (the GO term ID GO:0000166) from the HPRD data. As shown in Table 5, only 5 out of the 70 interactions involve a protein with an explicit annotation of 'Nucleotide binding'. The remaining 65 interactions were inferred from the Gene Ontology by finding a protein annotated with a more specialized term such as 'ATP binding', 'FAD binding' or 'GTP binding' than 'Nucleotide binding' (see Figure 2 for the partial view of the Gene Ontology of molecular function).

**Table 5:** Ontology-based search with 'Nucleotide binding' GO term.

Query protein		Partner protein	
HPRD_ID	Function	HPRD_ID	Function
HPRD_02944	ATP binding	HPRD_02431	Acyltransferase activity
HPRD_01368	ATP binding	HPRD_02147	ATP binding
HPRD_01368	ATP binding	HPRD_02300	ATPase activity
HPRD_02147	ATP binding	HPRD_12171	Catalytic activity
HPRD_01368	ATP binding	HPRD_02110	Extracellular matrix structural constituent
HPRD_02944	ATP binding	HPRD_02682	GTPase activity
HPRD_09468	ATP binding	HPRD_08986	Phospholipase activity
HPRD_01368	ATP binding	HPRD_02610	Protein binding
HPRD_02944	ATP binding	HPRD_03913	Protein binding
HPRD_09468	ATP binding	HPRD_01496	Protein serine/threonine kinase activity
HPRD_09468	ATP binding	HPRD_02619	Protein serine/threonine kinase activity
HPRD_09468	ATP binding	HPRD_03479	Protein serine/threonine kinase activity
HPRD_05802	ATP binding	HPRD_04066	Protein serine/threonine kinase activity
HPRD_09468	ATP binding	HPRD_05428	Protein serine/threonine kinase activity
HPRD_05802	ATP binding	HPRD_02963	Receptor activity
HPRD_09468	ATP binding	HPRD_01158	Structural constituent of myelin sheath
HPRD_02147	ATP binding	HPRD_01235	Transcription regulator activity
HPRD_09468	ATP binding	HPRD_00591	Translation regulator activity
HPRD_09468	ATP binding	HPRD_06774	Translation regulator activity
HPRD_09468	ATP binding	HPRD_06802	Translation regulator activity
HPRD_09468	ATP binding	HPRD_09084	Translation regulator activity
HPRD_01368	ATP binding	HPRD_03051	Transporter activity
HPRD_16742	FAD binding	HPRD_11762	DNA binding
HPRD_16742	FAD binding	HPRD_02171	Hydrolase activity
HPRD_04100	GTP binding	HPRD_07135	Acyltransferase activity
HPRD_04100	GTP binding	HPRD_01721	Auxiliary transport protein activity
HPRD_04100	GTP binding	HPRD_01722	Auxiliary transport protein activity
HPRD_04100	GTP binding	HPRD_04738	GTP binding
HPRD_04738	GTP binding	HPRD_06716	GTP binding
HPRD_11978	GTP binding	HPRD_00743	GTPase activity
HPRD_11978	GTP binding	HPRD_00766	GTPase activity
HPRD_04100	GTP binding	HPRD_03297	GTPase activity
HPRD_10360	GTP binding	HPRD_03297	GTPase activity
HPRD_04100	GTP binding	HPRD_12228	GTPase activity
HPRD_04738	GTP binding	HPRD_12228	GTPase activity
HPRD_10360	GTP binding	HPRD_06692	GTPase activity
HPRD_10360	GTP binding	HPRD_08555	GTPase activity
HPRD_11978	GTP binding	HPRD_09191	GTPase activity
HPRD_11978	GTP binding	HPRD_09973	GTPase activity
HPRD_11978	GTP binding	HPRD_11820	GTPase activity
HPRD_10360	GTP binding	HPRD_04398	Protein binding
HPRD_11978	GTP binding	HPRD_01265	Protein serine/threonine kinase activity
HPRD_06419	GTP binding	HPRD_03384	Protein serine/threonine phosphatase activity
HPRD_04100	GTP binding	HPRD_06288	RNA binding
HPRD_04738	GTP binding	HPRD_01853	Structural constituent of cytoskeleton
HPRD_04100	GTP binding	HPRD_01451	Structural molecule activity
HPRD_06419	GTP binding	HPRD_01859	Transcription factor activity
HPRD_06419	GTP binding	HPRD_16515	Transcription regulator activity
HPRD_04100	GTP binding	HPRD_03967	Ubiquitin-specific protease activity
HPRD_04738	GTP binding	HPRD_03967	Ubiquitin-specific protease activity
HPRD_09704	Nucleotide binding	HPRD_03356	Signal transducer activity
HPRD_09704	Nucleotide binding	HPRD_16544	Transcription factor activity
HPRD_09704	Nucleotide binding	HPRD_03221	Transcription regulator activity
HPRD_13115	Nucleotide binding	HPRD_03015	Unknown
HPRD_09704	Nucleotide binding	HPRD_04484	Unknown

In HPRD the ontology-based search found 70 interactions involving a protein annotated with 'Nucleotide binding'. Only 5 interactions have a protein with an explicit annotation of 'Nucleotide binding'. The remaining 65 interactions were inferred by finding a protein annotated with a more specialized term such as 'ATP binding', 'FAD binding' or 'GTP binding'. Due to space limit, 15 interactions (7 self-interactions and 8 interactions involving a protein with unknown function) are not shown here.

Networks 7-11 of Figure 3 represent the 31 interactions found by the ontology-based search with the query of 'ATP binding' (GO:0005524). 'ATP binding' is at the lower level than 'Nucleotide binding' in the ontology hierarchy, and therefore it is quite reasonable that the search results with 'ATP binding' are exclusively included in the search results with 'Nucleotide binding'. On the contrary, the ID-matching search found only 5 interactions (networks 5-6) with the query of 'Nucleotide binding' and missed the remaining 65 interactions. But with the query of 'ATP binding' the ID-matching search found the same 31 interactions (networks 7-11) as those found by the ontology-based search.

The search engine also allows the user to specify multiple conditions on the query protein. Table 6 shows the search results by the two search methods when the user specifies two GO terms, 'protein metabolic process' (GO:0019538) and 'cytoplasm' (GO:0005737), as conditions on the query protein. The ontology-based search found more interactions than the ID-matching search for all queries in the table.

**Table 6:** Example of searching protein-protein interactions by specifying multiple GO terms on the query protein.

Multiple GO terms		Search method	
Query protein		ID-matching search	Ontology-based search
Biological process	Cellular component		
GO:0019538	GO:0005737	1994 (5.22%)	3062 (8.02%)
GO:0019538	GO:0005576	753 (1.97%)	769 (2.01%)
Molecular function	Cellular component		
GO:0003700	GO:0005737	576 (1.51%)	592 (1.55%)
GO:0003700	GO:0005576	6 (0.02%)	103 (0.27%)

Search results when two GO terms are specified on the query protein, one for the biological process and another for the cellular component of the query protein.

**Figure 4:**
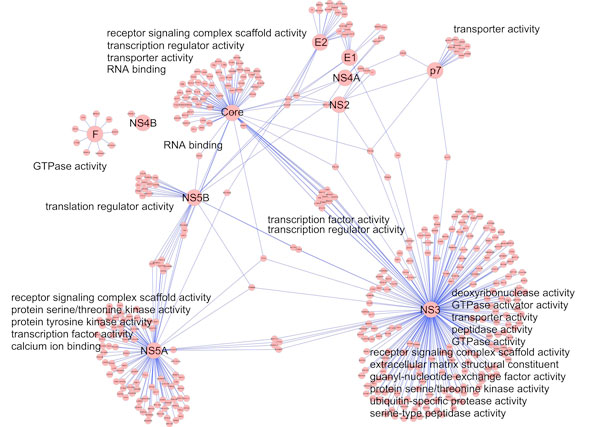
**Interaction network of HCV-human proteins**. The network contains HCV proteins (core, E1, E2, NS2, NS3, NS4A, NS4B, NS4A, NS5B, F and p7) and the human proteins interacting with them. The interaction data was obtained from a literature [19] and the network was visualized by Cytoscape [18]. The GO annotations for the HCV proteins and human proteins in the network are available at [21].

The implementation of the ontology-based search engine is not complete yet and being expanded to support various query types. For example, it will allow the user to search interactions by specifying GO terms both on the query and interaction partner proteins or by specifying multiple GO terms on the interaction partner protein. Figure 4 shows an interaction network between hepatitis C virus (HCV) and human proteins, which was constructed with the interaction data from a literature [19]. Since the current search engine has the modified Gödel number representation for human proteins only, it cannot find interaction partners in other species yet. However, the search engine will be expanded to retrieve interactions between human proteins and other types of proteins. The Gene Ontology annotations for the HCV proteins and human proteins in the network of Figure 4 are available at [21].

## Conclusion

We have developed a new method for representing protein-protein interactions and the Gene Ontology (GO) using modified Gödel numbers. This representation is hidden from users but enables a search engine to efficiently find protein-protein interactions in a biologically meaningful way. A prototype of the search engine is available at [21]. The search engine can find all interactions involving the query protein in almost real-time since the interaction partners of the query protein can be found unambiguously by prime factorization of the modified Gödel numbers representing the query protein and the search conditions.

The OWL Web Ontology Language [20] was established, but there have been no databases of protein-protein interactions that can process queries like "find every protein p with function f" or "for every protein p with function f, find the interaction partners of p". To the best of our knowledge, the ontology-based search engine presented in this paper is the first one that can deal with such queries. This paper presented preliminary results of the ontology-based search engine, and it is being expanded into a full-featured search engine.

## Competing interests

The authors declare that they have no competing interests.

## Authors' contributions

Byungkyu Park conceived the idea of using modified Gödel numbers, implemented the system and prepared the first draft of the manuscript. Kyungsook Han supervised the work and rewrote the manuscript.
